# Protective effects of ginseng on neurological disorders

**DOI:** 10.3389/fnagi.2015.00129

**Published:** 2015-07-16

**Authors:** Wei-Yi Ong, Tahira Farooqui, Hwee-Ling Koh, Akhlaq A. Farooqui, Eng-Ang Ling

**Affiliations:** ^1^Department of Anatomy, National University of SingaporeSingapore, Singapore; ^2^Neurobiology and Ageing Research Programme, National University of SingaporeSingapore, Singapore; ^3^Department of Molecular and Cellular Biochemistry, The Ohio State UniversityColumbus, OH, USA; ^4^Department of Pharmacy, National University of SingaporeSingapore, Singapore

**Keywords:** ginseng, ginsenoside, neuroprotection, neurodegeneration, neurons, glial cells, brain

## Abstract

Ginseng (Order: Apiales, Family: Araliaceae, Genus: *Panax*) has been used as a traditional herbal medicine for over 2000 years, and is recorded to have antianxiety, antidepressant and cognition enhancing properties. The protective effects of ginseng on neurological disorders are discussed in this review. Ginseng species and ginsenosides, and their intestinal metabolism and bioavailability are briefly introduced. This is followed by molecular mechanisms of effects of ginseng on the brain, including glutamatergic transmission, monoamine transmission, estrogen signaling, nitric oxide (NO) production, the Keap1/Nrf2 adaptive cellular stress pathway, neuronal survival, apoptosis, neural stem cells and neuroregeneration, microglia, astrocytes, oligodendrocytes and cerebral microvessels. The molecular mechanisms of the neuroprotective effects of ginseng in Alzheimer’s disease (AD) including β-amyloid (Aβ) formation, tau hyperphosphorylation and oxidative stress, major depression, stroke, Parkinson’s disease and multiple sclerosis are presented. It is hoped that this discussion will stimulate more studies on the use of ginseng in neurological disorders.

## Introduction

Ginseng (Order: Apiales, Family: Araliaceae, Genus: *Panax*) roots, stems, and leaves have been used as traditional herbal medicine for over 2000 years. In Korea, China and Japan, ginseng is the most valuable of all medicinal herbs. Its anti-anxiety, antidepressant and cognition-enhancing effects has been recorded by Shi-Zhen Li in Ben Cao Gang Mu (本草纲目) which is the most comprehensive pre-modern herbal text, compiled during the Ming Dynasty in China. *Panax ginseng* (人參) is mostly cultivated in Korea, the Manchuria region of China (“dong bei”) and the coastal region of Siberia due to its sensitivity to temperature and soil conditions. *Panax quinquefolium* L (American ginseng, 西洋參) is cultivated in southern Canada and the USA, and *Panax notoginseng* (田七) is grown in the Yunnan and Guangxi provinces of China. These three herbs represent the most extensively investigated species of *Panax*. The latter means “cure all”, and constituents of ginseng root produce adaptogenic, restorative, immunomodulatory, vasodilatory, anti-inflammatory, antioxidant, anti-aging, anticancer, anti-fatigue, anti-stress and anti-depressive effects in rodents and humans (Attele et al., 1999; Shin et al., 2000; Chang et al., 2003; Choo et al., 2003; Cheng et al., 2005; Wang et al., 2009). The bioactive ingredients in ginseng root include more than 60 ginsenosides, e.g., Rb1, Rb2, Rb3, Rc, Rd, Re, Rg1, Rg2, and Rg3, as well as polysaccharides, fatty acids, oligopeptides and polyacetylenic alcohols (Qi et al., 2010). The purpose of this review is to discuss the effects of ginseng on the normal brain, and its protective effects in neurological disorders such as Alzheimer’s disease (AD), major depression, stroke, Parkinson’s disease and multiple sclerosis. It is hoped that will stimulate more studies on the use of ginseng in neurological disorders.

## Intestinal Metabolism and Actions of Ginseng

**Figure 1:**
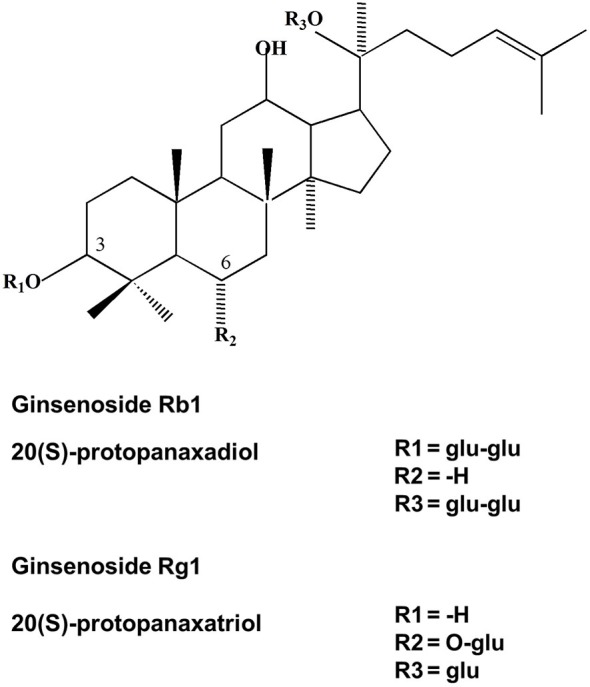
**Chemical structure of ginsenosides Rb1 and Rg1**. Ginsenoside Rb1 is an example for 20 (S)-PD and ginsenoside Rg1 is an example for 20 (S)-PT type of ginsenosides.

Ginsenosides are triterpene saponins that have a common four ring hydrophobic steroid-like structure with sugar moieties (monomeric, dimeric, or trimeric) attached mainly at carbons 3, 6 and 20 (Zhu et al., 2004; Nah et al., 2007). Ginsenosides are divided into two different structural classes: the 20 (S)-protopanaxadiol (PD) group of ginsenosides Ra1, Ra2, Ra3, Rb1, Rb2, Rb3, Rc, Rd, Rg3 and Rh2 and the 20 (S)-protopanaxatriol (PT) group ginsenosides Re, Rf, Rg1, Rg2, and Rh1 (Baek et al., 2012). Figure 1 shows the chemical structures of the two ginsenosides Rb1 and Rg1. In addition, several new ginsenosides such as 25-OH-pPD and 25-OH-pPT have been isolated and characterized from the fruit. 25-OH-pPD shows strong cancer preventing activity* in vitro* (Wang et al., 2007). Four malonyl derivatives of ginsenosides Rb1, Rb2, Rc and Rd have also been described (Fuzzati, 2004). The different sugar moieties in ginsenosides have been proposed to provide specificity for the therapeutic effects of ginsenosides (Zhu et al., 2004; Nah et al., 2007). The most commonly studied ginsenosides are Rb1, Rg1, Rg3, Re, and Rd. Intact ginsenosides are absorbed only from the intestines (the absorption rate is as low as 1 to 3.7%) and most ginsenosides are metabolized in the stomach (acid hydrolysis) and in the intestine (bacterial hydrolysis) or transformed to other ginsenosides. *In vitro* studies have indicated that ginsenoside Rb1 is metabolized by human intestinal bacteria. The metabolism is initiated by ginsenoside Rd (Rd) pathway at the C-20 glucose (Rb1 → Rd → ginsenoside F2 (F2) → Compound K (Cpd K) (Hasegawa et al., 1997; Hasegawa and Uchiyama, 1998; Tawab et al., 2003; Hasegawa, 2004; Bae et al., 2006; Qian et al., 2006; Chen et al., 2008b; Hou et al., 2012; Jung et al., 2012; Zhao et al., 2012). Collective evidence suggests that the *metabolism and transformation of intact ginsenosides* is an important process, playing an important role not only in bioavailability but also in the potential health benefits of ginseng (Chen et al., 2008a), such as anti-tumor (Xu et al., 2007; Lee et al., 2009), antioxidant, anti-inflammatory (Keum et al., 2003; Bae et al., 2006), anti-fatigue and angio-suppressive effects (Yue et al., 2006). Thus, ginseng is a natural remedy which not only enhances phagocytosis, natural killer cell activity, psychological function, cardiac function, and exercise performance, but also improves immune function by enhancing the production of interferons and increasing resistance to stress (Kiefer and Pantuso, 2003).

Many of the beneficial effects of ginseng are mediated through modulation of enzymes involved in signal transduction processes (Table 1). Administration of ginseng extract over 10 days inhibited peroxisome proliferator-activated receptor-α function, and decreased the expression of several genes involved in lipid and lipoprotein metabolism, but increased serum concentrations of total cholesterol, high-density lipoprotein cholesterol and triglycerides (Yoon et al., 2003). Jiannaoning, a Chinese herbal formula that includes ginseng leaves, improved memory function in rats subjected to cerebral ischemia (Song et al., 2006). It is proposed that Jiannaoning acts by regulating the levels of proinflammatory cytokines—interleukin-2 (IL-2), IL-6 and neuropeptide Y in the rat brain. Moreover, ginsenosides inhibited the tumor necrosis factor-alpha (TNF-α) induced phosphorylation of IkappaB-α (IκB-α) kinase (IKK) and the subsequent phosphorylation and degradation of IκB-α. TNF-α-induced phosphorylation of mitogen-activated protein kinase kinase 4 (MKK4) and subsequent activation of the c-Jun N-terminal kinases activator-protein 1 (JNK-AP-1) pathway was also suppressed (Choi et al., 2007). Ginsenosides from ginseng leaves and stems upregulated the level of glucocorticoid receptor (GR) in the brain cytosol of heat-injured rats (Li et al., 2006). Interactions of ginsenoids or glucocorticoids with GR triggered cellular responses involving activation of phosphatidylinositol-3 kinase (PI3K)/Akt pathway and increased nitric oxide (NO) production in human umbilical vein endothelial cells (Lee et al., 1997; Dancey, 2004). Ginsenosides may also regulate ion channels via their ligand-binding sites or channel pore sites (Nah et al., 2007). They inhibited voltage-dependent Ca^2+^, K^+^, and Na^+^ channel activities in a stereospecific manner (Liu et al., 2010) and blocked some subtypes of nicotinic acetylcholine (ACh) and 5-hydroxytryptamine type 3 receptors (Chen et al., 2010). Ginsenosides facilitated neurotransmission in the brain (Xue et al., 2006; Liu et al., 2010) but inhibited glutamate excitotoxicity (Kim et al., 2002). In addition, ginsenosides have been reported to improve central cholinergic function, and are used to treat memory deficits in humans (Rudakewich et al., 2001). Ginsenosides increased levels of dopamine and norepinephrine in the cerebral cortex (Itoh et al., 1989) and have beneficial effects on attention, cognitive processing, sensorimotor function and auditory reaction time in healthy subjects (D’Angelo et al., 1986).

**Table 1:** **Effect of ginsenosides on enzyme activities**.

Enzyme	Effect	Reference
Superoxide dismutase	Stimulation	Fu and Ji (2003) and Sohn et al. (2013)
Glutathione peroxidase	Stimulation	Fu and Ji (2003) and Sohn et al. (2013)
Na^+^, K^+^-ATPase	Inhibition	Chen et al. (2009)
Phosphoinositol-3-kinase (Ptd Ins 3K)/Akt-dependent extracellular signal-regulated kinase 1/2	Stimulation	Kim et al. (2007)
Endothelial nitric oxide synthase	Stimulation	Kim et al. (2007)

## Bioavailability of Ginseng

The oral bioavailability of ginsenosides is very low. The poor bioavailability of ginsenosides is not only because of low membrane permeability and active biliary excretion, but also due to biotransformation (Liu et al., 2009). As mentioned above, deglycosylated products of ginsenosides are formed by their bacterial metabolism in the gut lumen. These products are more permeable and bioactive than glycosylated ginsenosides (Hasegawa and Uchiyama, 1998). Intestinal bacterial metabolites of ginseng are absorbed into the bloodstream. Quantitative and statistical analysis of ginsenosides in plasma indicates that protopanaxadiol (PD) saponins exhibit higher concentration and longer half-life than protopanaxatriol (PT) saponins (Zhang et al., 2014). The mean values of half-lives of the ginsenosides Rg1, Re, Rb1, Rb2/b3, Rc and Rd are 15.26, 2.46, 18.41, 27.70, 21.86 and 61.58 h; while their peak concentrations are 7.15, 2.83, 55.32, 30.22, 21.42, 8.81 μg/L respectively (Zhang et al., 2014). Studies on concentrations of ginsenosides in the brain at different time intervals after dosing show that ginsenosides enter into the brain rapidly, but concentrations decline rapidly with time. Ginsenosides with higher concentrations in the brain are Rg1, Re, Rb1 and Rc (Zhang et al., 2014). Rg1 and Re may be the main components directly affecting CNS neurons, due to their better brain distribution. On the other hand, ginsenosides of PD saponins may protect the brain mostly through a peripheral effect, due to their longer time in the circulation.

## Molecular Mechanisms of Effects of Ginseng on the Brain

### Effect on Glutamatergic Neurotransmission

Ginsenosides have a general *stimulatory* effect on the brain (Radad et al., 2011). Ginsenoside Rb1 increased glutamate release in neurons via the PKA-dependent signaling pathway, whereas ginsenoside Rg1 induced glutamate release in a calcium/calmodulin-dependent protein kinase II (CaMKII) dependent manner (Liu et al., 2010). Ocotillol is a derivative of pseudoginsenoside-F11, a ginsenoside found in American ginseng, and displayed excitatory effect on spontaneous action potential firing and depolarized the membrane potential of mitral cells. This effect was abolished by the α-amino-3-hydroxy-5-methyl-4-isoxazolepropionic acid (AMPA)/kainate receptor antagonist 6-cyano-7-nitroquinoxaline-2,3-dione (CNQX) and the N-methyl-D-aspartate receptor (NMDAR) receptor antagonist D-AP5, suggesting a role for glutamatergic transmission in the effects of ocotillol (Wang et al., 2011b). Transmission of action potentials transmission in the somatosensory cortex was facilitated by ginsenoside Rb3 perfusion (Cui et al., 2012). Ginsenoside Rg1 supplementation improved the performance of aged mice in behavioral tests and significantly upregulated the expression of synaptic plasticity-associated proteins in the hippocampus, including synaptophysin, NMDAR subunit 1, postsynaptic density protein 95 (PSD95) and CaMKII alpha via the mammalian target of rapamycin (mTOR) pathway (Yang et al., 2014). Notoginsenoside R1, a major saponin isolated from *Panax notoginseng*, increased membrane excitability of CA1 pyramidal neurons in hippocampal slices by lowering the spike threshold, possibly through inhibition of voltage-gated K^+^ currents. It also reversed Aβ_1–42_ oligomer-induced impairments in long-term potentiation (Yan et al., 2014). Other studies showed that ginsenoside Rb1 inhibited the activity of L-type voltage gated calcium channels, without affecting N-type or P/Q-type Ca^2+^ channels in hippocampal neurons (Lin et al., 2012a). Gintonin is a newly identified compound from ginseng that is found to activate G protein-coupled lysophosphatidic acid (LPA1) receptors with high affinity (Hwang et al., 2012a; Nah, 2012). Gintonin stimulated neurotransmitter release (Hwang et al., 2015) and promoted synaptic enhancement via LPA1 receptor activation in central synapses (Park et al., 2015). Gintonin contains two components: “ginseng major latex-like protein 151” (GLP) and ginseng ribonuclease-like storage protein; and recent studies have determined the structure of ginseng major latex-like protein 151 and its proposed lysophosphatidic acid binding mechanism (Choi et al., 2015). The LPA1 receptor is localized in dendritic spines of cultured hippocampal neurons (Pilpel and Segal, 2006) that are sites of glutamatergic synapses, and further study is necessary to elucidate a possible role of gintonin and LPA receptor signaling, in the CNS effects of ginseng.

### Effect on Monoamine Neurotransmission

Ginsenosides could have effects on monoamine signaling. Total saponins extracted from *Panax notoginseng* increased the levels of 5-hydroxytryptamine, dopamine and noradrenaline, suggesting that it may have antidepressant effects by modulation of brain monoamine levels (Xiang et al., 2011).

### Effect on Estrogen Signaling

Improved object recognition and decreased immobility time in the forced swim test have been reported following ginsenoside Rb1 treatment. Pre-treatment with an estrogen receptor antagonist clomiphene blocked these effects, indicating that they are dependent on the estrogen receptor (Hao et al., 2011). Red ginseng also prevented downregulation of the ERβ estrogen receptor in immobilization-stressed mice (Kim et al., 2013b).

### Effect on Nitric Oxide Production

Ginsenosides have been shown in many studies to *inhibit* inducible nitric oxide synthase (iNOS). Rg3 at 20 and 30 mg/kg oral doses modulated an increase in TNF-α, IL-1β and IL-6 mRNA after lipopolysaccharide (LPS) injection in mice (Park et al., 2012b). Protective effects of Re treatment occurred via the phospho-p38, iNOS, and cyclooxygenase-2 (COX-2) signaling pathways in LPS stimulated BV-2 microglial cells (Lee et al., 2012). A bacterial metabolite of Rg5 which is the main constituent of heat-processed ginseng, Rh3, also decreased the expression of iNOS and TNF-α and IL-6, and increased the expression of heme oxygenase −1(HO-1) in LPS stimulated BV-2 microglial cells (Lee et al., 2015). As with iNOS, neuronal nitric oxide synthase (nNOS) is *inhibited* by ginseng. Ginseng saponins are transformed by intestinal microflora, and a product Rh2 decreased the expression of nNOS in the hippocampus, while another product Rg3 decreased its expression in the neocortex (Jang et al., 2004). Ginsenoside Rg1 has neuroprotective effects on ischemia-reperfusion injury in cultured hippocampal cells by blocking calcium influx into neuronal cells and decreasing nNOS activity (He et al., 2014). In contrast to iNOS, and nNOS, ginsenosides appear to *stimulate* endothelial nitric oxide synthase (eNOS) and may have beneficial effects on the circulation. Ginsenoside-Rg1 increased the phosphorylation of GR, PI3K, Akt/PKB and eNOS, leading to NO production in human umbilical vein endothelial cells. Knockdown of GR modulated the Rg1 induced NO production. Results indicate that Rg1 can act as an agonist ligand for GR, and that activated GR can induce rapid NO production from eNOS via the PI3K/Akt pathway (Leung et al., 2006).

### Effect on the Keap1/Nrf2 Adaptive Cellular Stress Pathway (Figure 2)

**Figure 2:**
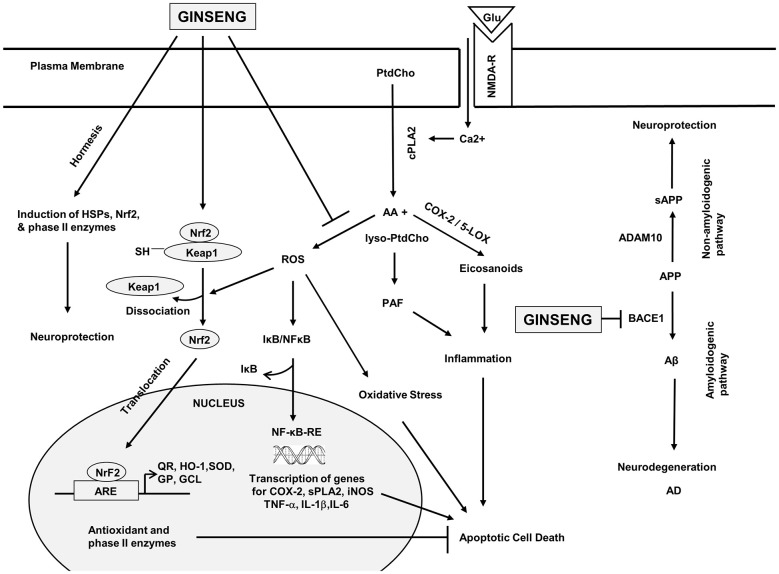
**Hypothetical diagram showing the effects of ginseng on signal transduction processes in the brain**. phosphatidylcholine (PtdCho); lyso-phosphatidylcholine (lyso-PtdCho); cytosolic phospholipase A2 (cPLA2); arachidonic acid (AA); platelet activating factor (PAF); reactive oxygen species (ROS); Nuclear factor-kappa B (NF-κB); nuclear factor erythroid 2-related factor 2 (Nrf2); cyclooxygenase-2 (COX-2); lipoxygenase (LOX); secretory phospholipase A2 (sPLA2); inducible nitric oxide synthase (iNOS); tumor necrosis factor-α (TNF-α); interleukin-1β (IL-1β); interleukin-6 (IL-6); kelch-like erythroid Cap “n” Collar homologue-associated protein 1 (Keap1); NFE2-related factor 2 (Nrf2); antioxidant response element (ARE); quinine oxidoreductase (QR); hemeoxygenase 1 (HO-1); superoxide dismutase (SOD); glutathione peroxidase (GP); γ-glutamylcysteine ligase (γ-GCL); heat shock proteins (HSPs); amyloid precursor protein (APP); soluble amyloid precursor protein (sAPP); β-amyloid (Aβ); α secretase (ADAM10); and β-secretase (BACE1). This diagram is based on information provided in Choi et al. (2010), Jung et al. (2010), Li et al. (2010), Ye et al. (2011b), Hwang et al. (2012b), Karpagam et al. (2013) and Wang et al. (2013c).

This is an important adaptive pathway in response to oxidative stress, and many studies have pointed to this pathway as a target of dietary phytochemicals (Reviewed in Lee et al., 2014). Under physiological conditions, Keap1 keeps the Nrf2 transcription factor in the cytoplasm, allowing it to be ubiquitinated and degraded by proteasomes; but when cells are exposed to neurodegenerative disease-mediated oxidative stress, a signal involving phosphorylation and/or redox modification in Keap1 blocks the enzymatic activity of the Keap1-Cul3-Rbx1 E3 ubiquitin ligase complex, leading to decrease in Nrf2 ubiquitination and degradation. As a result, free Nrf2 translocates into the nucleus, where it transactivates the antioxidant response element (ARE) of many cytoprotective genes (Figure 2). Rg1 pretreatment induced Nrf2 nuclear translocation and activation of the PI3K/Akt pathway, which could antagonize iron-induced increase in reactive oxygen species (ROS) and decrease in mitochondrial transmembrane potential in cultured neurons (Du et al., 2013). Likewise, ginsenoside Rh3 increased Nrf2 DNA-binding activity and level of sirtuin 1 (SIRT1) resulting in inhibition of NF-κB in cultured cells (Lee et al., 2015). Protopanaxtriol extracted from *Panax ginseng* increased Nrf2 entering the nucleus, and induced the expression of phase II antioxidant enzymes, including HO-1 and nicotinamide adenine dinucleotide phosphate (NADPH) quinone oxidase 1, after 3-nitropropionic acid induced injury (Gao et al., 2015). Notoginsenoside R1 isolated from *Panax notoginseng* increased nuclear translocation of Nrf2 and ARE activity, and upregulated the expression and activity of HO-1, NADPH quinone oxidase 1 and gamma-glutamylcysteine synthetase (γ-GCSc) in PC12 cells (Meng et al., 2014). Together, results indicate an important role of the Keap1/Nrf2 pathway in neuroprotective effects of ginseng.

### Effect on Neuronal Cell Survival

Oral administration of ginsenoside Rb1 significantly increased cell survival but not proliferation in the hippocampus, that may be related to its effects on learning and memory (Liu et al., 2011).

### Effect on Apoptosis

Ginsenoside Rg induced downregulation of calpain I and caspase-3 and attenuated neuronal apoptosis caused by cerebral ischemia-reperfusion injury (He et al., 2012). Ginsenoside Rb1 is reported to suppress the activation of endoplasmic reticulum stress-associated proteins including protein kinase RNA (PKR)-like ER kinase (PERK) and C/EBP homology protein (CHOP) and downregulation of Bcl-2 induced by high glucose (Liu et al., 2013). *Panax notoginseng* saccharides increased Bcl-2/Bax ratio and had anti-apoptotic and neuroprotective effects after cerebral ischemia in rats (Jia et al., 2014).

### Effect on Neural Stem Cells and Neuroregeneration

Ginsenoside Rb1 enhanced neurotrophin expression and induced differentiation of midbrain dopaminergic neurons (Hsieh and Chiang, 2014). In addition, Rg1 promoted neural stem cell differentiation through the cyclic adenosine monophosphate-protein kinase A (cAMP-PKA) and PI3K-AKT signaling pathways (Jiang et al., 2012a; Li et al., 2013). Ginsenoside Rd also increased neural stem cell proliferation (Lin et al., 2012b) and maintained neurogenesis after lead-induced neural injury (Wang et al., 2013b).* Panax notoginseng* saponins decreased the expression of the neurite inhibitory molecules Nogo-A and NgR after cerebral ischemia in rats, suggesting a role in axonal regeneration after injury (Liu et al., 2014).

### Effect on Microglia

Microglia are resident innate immune cells in the CNS, which are activated in response to a variety of stimuli, such as infection, traumatic brain injury, toxic metabolites, or autoimmunity. Ginsenoside Rh1 inhibited the expression of iNOS, COX-2, and pro-inflammatory cytokines while increasing the expression of anti-inflammatory IL-10 in LPS-stimulated microglia. These effects were attenuated by inhibition of HO-1 (Jung et al., 2010). In addition, ginsenoside Rb1 reversed the changes in several direct or indirect neuroinflammatory markers in the hippocampus (Wang et al., 2011a). Ginsenoside Rb1 also decreased the expression of TNF-α, down regulated IL-6 expression, inhibited the activation of NF-κB and modulated microglial activation after brain ischemia (Zhu et al., 2012). Ginsenoside Rg1 pre-treatment of LPS induced BV-2 microglial cells activated the phospholipase C signaling pathway and modulated the expression of iNOS, COX-2, TNF-α, IL-1β and NF-κB (Zong et al., 2012). A ginseng saponin metabolite, compound K (20-O-D-glucopyranosyl-20 (S)-PD), reduced the number of Iba1-positive activated microglia, and inhibited the expression of TNF-α and IL-1β in the brain after LPS-induced sepsis (Park et al., 2012a). Together, results indicate that ginseng has anti-neuroinflammatory effects by reducing activation of microglia.

#### Effect on Astrocytes

Ginsenoside Rd increased levels of phosphorylated protein kinase B (PKB/Akt) and phospho-ERK1/2 (p-ERK1/2) and expression of glutamate transporter-1 (GLT-1) in astrocyte cultures after oxygen-glucose deprivation. The effect of Rd on GLT-1 expression was abolished by inhibition of PI3K/AKT or ERK1/2 signaling pathways (Zhang et al., 2013). Moreover, total saponins in the leaves of *Panax notoginseng* reduced H_2_O_2_ induced cell death in primary rat cortical astrocytes, likely through nuclear translocation of Nrf2 and upregulation of antioxidant systems including HO-1 and glutathione S-transferase (Zhou et al., 2014b). In contrast to normal or hypoxic astrocytes, ginsenoside Rg3 showed anti-cancer activities in malignant astrocytes glioblastoma multiforme cells, by induction of apoptosis via the methyl ethyl ketone (MEK) signaling pathway (Choi et al., 2013).

#### Effect on Oligodendrocytes and Myelination

Administration of American ginseng* Panax quinquefolium* to mice at a dose of 150 mg/kg body mass resulted in improvement of demyelination scores after induction of experimental autoimmune encephalomyelitis (EAE; Bowie et al., 2012).

#### Effect on Cerebral Microvessels

Ginsenosides may be effective in inhibiting changes to the cerebral microvasculature after cerebral ischemia. Weinaokang, a preparation consisting of active compounds extracted from *Ginkgo biloba*, ginseng, and *Crocus sativus* (saffron) inhibited the translocation of G protein-coupled receptor kinase 2 from the cytosol to the cell membrane, and reduced extracellular-signal-regulated kinases (ERK1/2) phosphorylation and matrix metalloproteinase expression in cerebral microvessels (Zheng et al., 2010). Ginsenoside Rg1 also modulated increase in aquaporin 4 expression and blood brain barrier (BBB) disruption after cerebral ischemia in rats (Zhou et al., 2014a).

## Protective Effects of Ginseng on Neurological Disorders

### Neurochemical Aspects of Neurodegenerative, Neurotraumatic, and Neuropsychiatric Disorders

Neurodegenerative diseases include AD, Parkinson’s disease, Huntington’s disease, and amyotrophic lateral sclerosis. They are associated with progressive loss of cognitive function and motor disabilities with devastating consequences to the affected individual. Genetic factors, environmental factors and unhealthy lifestyle have been suggested to contribute to the pathogenesis of neurodegenerative processes (Farooqui, 2010; Seidl et al., 2014). Many neurodegenerative diseases are accompanied by oxidative stress and neuroinflammation, and associated with increased generation of lipid mediators, abnormal protein aggregation, slow excitotoxicity, loss of synapses and disintegration of neural networks, leading to failure of neurological functions (Jellinger, 2009; Farooqui, 2010). Neurotraumatic diseases are caused by metabolic or mechanical insult to the brain and spinal cord. They include cerebral ischemia or stroke, spinal cord injury and traumatic brain injury. Neurochemical events in neurotraumatic diseases include the release of glutamate, overstimulation of glutamate receptors, rapid calcium influx, activation of cytosolic phospholipase A_2_ (cPLA_2_), phospholipase C, COX-2 and NOS, induction of oxidative stress and neuroinflammation (Farooqui, 2010). Neuropsychiatric disorders include both neurodevelopmental disorders and behavioral or psychological difficulties such as depression, schizophrenia, and bipolar disorders. Impairment of cognitive processes results in behavioral symptoms such as abnormal thoughts or actions, delusions, and hallucinations. They involve abnormalities in the cerebral cortex, ventral striatum and other components of the limbic system (Gallagher, 2004).

### Alzheimer’s Disease (Table 2)

#### Cellular Changes Induced by Ginseng

AD is the most common form of dementia, a general term for memory loss and other intellectual abilities serious enough to interfere with daily life. It is characterized by the presence of extracellular plaques consisting of aggregated Aβ peptides and neurofibrillary tangles that contains hyperphosphorylated tau protein, as well as cerebral amyloid angiopathy, due to Aβ deposition around the walls of arterioles or capillaries in the brain (Reviewed in Cheng et al., 2005). Aβ peptides are generated from amyloid precursor protein (APP) by β-secretase (BACE1) and γ-secretase activities, and are generally considered to be toxic to neurons, while neurofibrillary tangles interfere with cellular transport processes. Treatment with *Panax notoginseng* flavonol glycosides inhibited the aggregation of Aβ in a dose-dependent manner and modulated the increase in Ca^2+^ and cell death triggered by Aβ in cultured neurons. These flavonol glycosides also reduced memory impairment in a passive avoidance task in scopolamine-treated rats (Choi et al., 2010). Treatment of SH-SY5Y neuroblastoma cells with gintonin, a novel LPA1 receptor activating ligand decreased Aβ1–42 release, and attenuated Aβ1–40 induced toxicity. In addition, long-term oral administration of gintonin attenuated amyloid plaque deposition, and short- and long-term memory impairment in a mouse model of AD (Hwang et al., 2012b). Fermented ginseng also reduced Aβ1–42 levels in HeLa cells stably expressing the Swedish mutant form of APP and decreased memory impairment in mouse models of AD (Kim et al., 2013a).

**Table 2:** **Studies summarizing the effects of ginseng metabolites in neurological disorders**.

Neurological disorder	Ginseng metabolite	Mechanism	Reference
Stroke	GinsenosideRd, GS-Rd, Ginsenoside Rb1	Anti-inflammatory; inhibition of Ca2+ influx, and reduction in edema	Ye et al. (2011a,b)
AD	Ginsenoside CK, F1, Rh1, and Rh2; *Panax* *notoginseng* saponins	Inhibition of A**β** aggregation, inflammation, and antioxidant effects	Karpagam et al. (2013) and Huang et al. (2014)
PD	Ginseng extract G115	Inhibition of **α**-synuclein aggregation	Van Kampen et al. (2014)
EAE and MS	Ginsenoside Rd	Regulation of IFN-**γ** and IL-4	Zhu et al. (2014)
Depression	Ginsenosides 20 (S) protopanaxadiol, Rg1, and Rb1	Antidepressant Upregulation of BDNF	Xu et al. (2010) and Wang et al. (2013c)

#### Effect on Aβ Formation

Ginseng may have beneficial effects in AD by reducing the formation of Aβ oligomers (Figure 2). Chronic supplementation with ginsenosides for 8 months in the drinking water modulated age-related memory impairment in mice (Zhao et al., 2011). *Panax notoginseng* saponins reduced brain APP mRNA levels and improved learning and memory in senescence-accelerated mice (Zhong et al., 2011), and daily consumption of American ginseng helped preserve cognitive functions in senescence-accelerated mice (Shi et al., 2012). Moreover, aged transgenic AD mice overexpressing APP/Aβ treated with ginsenoside Rg1 showed marked decrease in cerebral Aβ levels, reversal of neuropathological changes and protection of spatial learning abilities and memory (Fang et al., 2012). Ginsenosides CK, F1, Rh1 and Rh2 from *Panax ginseng* were found to have potential BACE1 inhibitory activities (Karpagam et al., 2013); and *Panax notoginseng* saponins increased non-amyloidogenic processing of APP by increasing α-secretase activity, and decreased amyloidogenic processing by decreasing BACE1 expression (Huang et al., 2014). Ginsenoside Rh2 improved learning and memory performance, and reduced the number of senile plaques in brains of Tg2576 mice (Qiu et al., 2014). Results suggest neuroprotective effects of ginsenosides by reducing Aβ levels via stimulation of α-secretase activity and inhibition of β-secretase activity.

#### Effect on tau Phosphorylation

Ginseng may also have a beneficial effect in AD by reducing tau hyperphosphorylation and neurofibrillary tangle formation. Total ginsenoside extracts from stems and leaves of *Panax ginseng* enhanced the phosphatase activity of purified calcineurin. This could be useful in AD, since inhibition of calcineurin leads to tau hyperphosphorylation at multiple sites in AD brains (Tu et al., 2009). Treatment with ginsenoside Rd cultured cortical neurons or AD rats (10 mg/kg for 7 days) reduced okadaic acid-induced neurotoxicity and tau hyperphosphorylation by enhancing the activities of protein phosphatase 2A (PP2A; Li et al., 2011a). Ginsenoside Rb1 reversed aluminum exposure-induced decreased PP2A level and tau phosphorylation in the cortex and hippocampus (Zhao et al., 2013). Ginsenoside Rg1 (20 mg/kg) reversed memory impairments induced by okadaic acid by decreasing levels of phosphorylated tau and suppressing the formation of Aβ in the brains of rats (Song et al., 2013). Results suggest neuroprotective effect of ginsenosides by reducing tau hyperphosphorylation.

#### Effect on Reactive Oxygen and Nitrogen Species

Ginsenosides are reported to have antioxidant effects. Rg1 is a potential regulator of hypoxia-inducible factor-1α (HIF-1α), and could act via this transcription factor to improve cell survival, angiogenesis and neurogenesis (Tang et al., 2011). Oral treatment with pseudoginsenoside-F11 (PF11), a component of *Panax quinquefolium* increased the activity of antioxidant enzymes superoxide dismutase (SOD) and glutathione peroxidase (GSH-Px), and is associated with improved learning and memory in a mouse model of AD (Wang et al., 2013c). Ginsenoside Rg1 treatment modulated Aβ_25–35_ induced mitochondrial dysfunction, inhibition of HIF-1α expression and increase in reactive nitrogen species and protein nitrotyrosination in human endothelial cells (Yan et al., 2013).

#### Effect on Cholinergic and Neurotrophic Signaling

Loss of ACh is found in the brain in AD. Ginsenoside Rg5 found in *Panax ginseng* modulated cognitive dysfunction and neuroinflammation in the brains of streptozotocin-induced memory impaired rats. Acetylcholinesterase (AChE) activity was reduced, while choline acetyltransferase (ChAT) activity was increased in the cortex of Rg5 treated rats (Chu et al., 2014). Ginsenosides Re and Rd also induced the expression of ChAT/VAChT and increased the level of ACh in Neuro-2a cells (Kim et al., 2014).

#### Clinical Studies on Use of Ginseng in AD

Patients in a high-dose Korean Red Ginseng group (9 g/day) showed significant improvement on the Alzheimer’s Disease Assessment Scale (ADAS), Clinical Dementia Rating (CDR) after 12 weeks of Korean Red Ginseng therapy, when compared with those in the control group (Heo et al., 2008). In the long-term evaluation of the efficacy of Korean Red Ginseng in patients after 24 weeks, the Korean version of the Mini Mental Status Examination (K-MMSE) score remained without significant decline at the 48^th^ and 96^th^ weeks, and similar results were obtained with ADAS. Together, results point to long-term beneficial effects of Korean Red Ginseng in patients with AD (Heo et al., 2011).

### Major Depression (Table 2)

#### Preclinical Studies

Major depressive disorder or major depression is a mental disorder characterized by a pervasive and persistent low mood that is accompanied by low self-esteem and by a loss of interest or pleasure in normally enjoyable activities. Ginseng total saponins at doses of 50 and 100 mg/kg for 7 days reduced the immobility time in the forced swim test, which is a mouse model of depression. They also reversed the reduction in the sucrose preference index, decrease in locomotor activity, as well as prolongation of latency of feeding in a novel environment, in a chronic stress model of depression in rats (Dang et al., 2009). Ginseng saponins also had antidepressant effects in the corticosterone-induced mouse model of depression (Chen et al., 2014). Mice that received total saponins extracted from *Panax notoginseng* at doses of 10–1000 mg/kg daily for 1, 7, and 14 days showed decreased immobility time in the forced swim test. In the chronic mild stress model, chronic total saponin treatment (70 mg/kg) reversed depression-like behavior in rats. Levels of 5-hydroxytryptamine, dopamine, and noradrenaline were increased in the brains of treated rats, which could be the basis of the antidepressant effects (Xiang et al., 2011). Ginsenoside Rg1 upregulated BDNF signaling in the hippocampus, down regulated serum corticosterone levels, and reversed the decrease in dendritic spine density and hippocampal neurogenesis caused by chronic stress (Jiang et al., 2012b; Wang et al., 2013a). A post-metabolism compound and intestinal metabolite of ingested ginsenosides, PD, showed antidepressant effects in the tail suspension and forced swim tests, in the olfactory bulbectomy rat model of depression (Xu et al., 2010). Together with findings in the “Effect on Glutamatergic Neurotransmission” “Effect on monoamine Neurotransmission”, “Effect on Estrogen Signalling”, “Effect on Neural Stem Cells and Neuroregeneration” sections, they suggest that ginseng may have beneficial effects in major depression via multiple actions: (a) increasing AMPA receptor signaling, in a manner similar to AMPA receptor agonists or AMPAkines; (b) increasing the level of monoamines; (c) reducing stress related corticosteroid levels; and (d) increasing estrogen receptor signaling and BDNF, which are related to decreased neuronal death and improved neurogenesis.

#### Clinical Studies

A randomized, multicenter, double-blind parallel group study of 193 post-menopausal women treated with ginseng and 191 treated with placebo reported significant difference in depression scores in favor of ginseng compared with placebo (Wiklund et al., 1999). Clinical studies on 35 female outpatients aging from 18 to 65 years who were remitted from major depression with residual symptoms and given Korean Red Ginseng at a dose of 3 g/day reported significant decrease in depressive symptoms on the Depression Residual Symptom Scale (DRSS) and Montgomery-Åsberg Depression Rating over an 8-week period (Jeong et al., 2014). Another study of possible casual relation between hormones, energy sources, and minerals indicated that depression was related to serum lipids including cholesterol, and that fermented red ginseng had beneficial effects on depression by modulating this relation (Lee and Ji, 2014).

### Stroke

Stroke or cerebrovascular event is associated with a sudden loss of brain function, due to disturbance in blood supply to the brain. Ginsenoside Rd (10–50 mg/kg) significantly decreased infarct volume and was associated with a better neurological outcome after transient middle cerebral artery occlusion (MCAO) in rats (Ye et al., 2011a). Interestingly, ginsenoside Rd demonstrated neuroprotection even when administered 4 h after recirculation of transient MCAO or after onset of ischemic stroke induced by permanent MCAO (Ye et al., 2011b). mRNA and protein expression levels of non-selective cation channels such as transient receptor potential melastatin (TRPM) and acid sensing ion channels (ASIC) were significantly increased 24 h following MCAO, and these effects were attenuated by treatment with 10 mg/kg ginsenoside Rd (Zhang et al., 2012). High dose ginsenoside Rb1 treatment reduced neurological deficits, brain edema, BBB disruption and the number of terminal deoxynucleotidyl transferase dUTP nick end labeling (TUNEL) positive cells after hemorrhagic stroke (Li et al., 2010). Significant reduction in basilar artery vasospasm and lumen thickness was also observed after Rb treatment (Li et al., 2011b). Results suggest that neuroprotective effect of ginseng in stroke via inhibition of ion channels or modulation of vasospasm. It is, however, noted that ginseng may have a drug interaction with the anticoagulant warfarin that could lead to increased risk of bleeding, and this should be taken into consideration in patients who are on warfarin.

### Parkinson’s Disease

Parkinson disease is a neurodegenerative disorder affecting mainly the motor system, as a result of death of dopaminergic neurons in the substantia nigra. Ginsenosides investigated as neuroprotective agents for Parkinson’s disease are Rb1, Rg1, Rd and Re. These compounds exert neuroprotective effects through inhibition of oxidative stress and neuroinflammation, decrease in toxin-induced apoptosis and nigral iron levels, and regulation of N-methyl-D-aspartate receptor channel activity (reviewed by González-Burgos et al., 2014). Oral administration of the ginseng extract, G115 modulated tyrosine hydroxylase-positive cell loss in the substantia nigra, and reduced locomotor dysfunction in the 1-methyl-4-phenyl-1, 2, 3, 6-tetrahydropyridine (MPTP) and 1-methyl-4-phenylpyridinium (MPP^+^) models of Parkinson’s disease (Van Kampen et al., 2003). G115 also reduced dopaminergic cell loss, microgliosis, and accumulation of α-synuclein aggregates after chronic exposure to the dietary phytosterol glucoside, β-sitosterol β-d-glucoside or “BSSG model” of chronic Parkinson’s disease (Van Kampen et al., 2014). MPTP administration resulted in behavioral impairment, dopaminergic neuronal death, and increased Cdk5 and p25 expression but decreased p35 expression in the nigrostriatal system of mice, and these effects were modulated by Korean Red Ginseng (Jun et al., 2015). Panaxatriol saponins, the main constituents extracted from *Panax notoginseng* provided neuroprotection against loss of dopaminergic neurons and behavioral impairment after MPTP treatment (Luo et al., 2011). Together, results suggest that ginseng may have neuroprotective effects in Parkinson’s disease.

### Multiple Sclerosis

Ginsenoside Rd has been studied in an animal model of multiple sclerosis, EAE. Intraperitoneally administered ginsenoside Rd at 40 mg/kg/day reduced the permeability of the BBB, regulated the secretion of interferon-gamma and IL-4, and decreased the severity of EAE in mice (Zhu et al., 2014).

## Conclusions and Future Directions

Many ginsenosides have been isolated and characterized. The molecular mechanisms associated with ginsenosides involve scavenging free radicals, inhibition of inflammation, and prevention of excitotoxicity. Animal and cell culture studies have indicated that ginsenosides have different activities in both physiological and pathologic conditions. The structure activity relationship of ginsenosides has not been fully elucidated. However, it is becoming increasingly evident that ginsenosides produce neuroprotective effects by reducing free radical production and enhancing brain function. Studies involving each ginsenoside should include mechanisms of action, specificity, structure and function relationship, detailed pharmacokinetics and toxicity studies, and therapeutic studies in animal models and humans. Further studies are necessary to examine the effects of ginseng on metabotropic glutamate receptors and transporters, and the Keap1/Nrf2 adaptive cellular pathway. The neurological disorders for which there is most evidence from pre-clinical and a small number of clinical studies to benefit from ginseng are AD and major depression, and clinical trials are necessary to confirm the efficacy of ginseng and ginsenosides in the prevention and treatment of these, and possibly other neurological conditions.

## Conflict of Interest Statement

The authors declare that the research was conducted in the absence of any commercial or financial relationships that could be construed as a potential conflict of interest.

## References

[B1] Attele A. S. Wu J. A. Yuan C. S. (1999). Ginseng pharmacology: multiple constituents and multiple actions. Biochem. Pharmacol. 58, 1685–1693. 10.1016/S0006-2952(99)00212-9 10571242

[B2] Bae E. A. Kim E. J. Park J. S. Kim H. S. Ryu J. H. Kim D. H. (2006). Ginsenosides Rg3 and Rh2 inhibit the activation of AP-1 and protein kinase A pathway in lipopolysaccharide/interferon-gamma-stimulated BV-2 microglial cells. Planta Med. 72, 627–633. 10.1055/s-2006-931563 16673329

[B3] Baek S. H. Bae O. N. Park J. H. (2012). Recent methodology in ginseng analysis. J. Ginseng Res. 36, 119–134. 10.5142/jgr.2012.36.2.119 23717112 PMC3659581

[B4] Bowie L. E. Roscoe W. A. Lui E. M. Smith R. Karlik S. J. (2012). Effects of an aqueous extract of North American ginseng on MOG (35-55)-induced EAE in mice. Can. J. Physiol. Pharmacol. 90, 933–939. 10.1139/y2012-092 22720838

[B5] Chang Y. S. Seo E. K. Gyllenhaal C. Block K. I. (2003). Panax ginseng: a role in cancer therapy? Integr. Cancer Ther. 2, 13–33. 10.1177/1534735403251167 12941165

[B8] Chen R. J. Chung T. Y. Li F. Y. Lin N. H. Tzen J. T. (2009). Effect of sugar positions in ginsenosides and their inhibitory potency on Na^+^/K^+^-ATPase activity. Acta Pharmacol. Sin. 30, 61–69. 10.1038/aps.2008.6 19060914 PMC4006530

[B9] Chen Z. Lu T. Yue X. Wei N. Jiang Y. Chen M.. (2010). Neuroprotective effect of ginsenoside Rb1 on glutamate-induced neurotoxicity: with emphasis on autophagy. Neurosci. Lett. 482, 264–268. 10.1016/j.neulet.2010.07.052 20667501

[B6] Chen G. Yang M. Song Y. Lu Z. Zhang J. Huang H.. (2008a). Comparative analysis on microbial and rat metabolism of ginsenoside Rb1 by high-performance liquid chromatography coupled with tandem mass spectrometry. Biomed. Chromatogr. 22, 779–785. 10.1002/bmc.1001 18384066

[B7] Chen G. T. Yang M. Song Y. Lu Z. Q. Zhang J. Q. Huang H. L.. (2008b). Microbial transformation of ginsenoside Rb (1) by Acremonium strictum. Appl. Microbiol. Biotechnol. 77, 1345–1350. 10.1007/s00253-007-1258-4 18040682

[B137] Chen L. Dai J. Wang Z. Zhang H. Huang Y. Zhao Y. (2014). The antidepressant effects of ginseng total saponins in male C57BL/6N mice by enhancing hippocampal inhibitory phosphorylation of GSK-3β. Phytother. Res. 28, 1102–1106. 25165785 10.1002/ptr.5103

[B10] Cheng Y. Shen L. H. Zhang J. T. (2005). Anti-amnestic and anti-aging effects of ginsenoside Rg1 and Rb1 and its mechanism of action. Acta Pharmacol. Sin. 26, 143–149. 10.1111/j.1745-7254.2005.00034.x 15663889

[B13] Choi S. H. Hong M. K. Kim H. J. Ryoo N. Rhim H. Nah S. Y.. (2015). Structure of ginseng major latex-like protein 151 and its proposed lysophosphatidic acid-binding mechanism. Acta Crystallogr. D Biol. Crystallogr. 71, 1039–1050. 10.1107/s139900471500259x 25945569

[B11] Choi K. Kim M. Ryu J. Choi C. (2007). Ginsenosides compound K and Rh (2) inhibit tumor necrosis factor-alpha-induced activation of the NF-kappaB and JNK pathways in human astroglial cells. Neurosci. Lett. 421, 37–41. 10.1016/j.neulet.2007.05.017 17548155

[B14] Choi Y. J. Lee H. J. Kang D. W. Han I. H. Choi B. K. Cho W. H. (2013). Ginsenoside Rg3 induces apoptosis in the U87MG human glioblastoma cell line through the MEK signaling pathway and reactive oxygen species. Oncol. Rep. 30, 1362–1370. 10.3892/or.2013.2555 23783960

[B12] Choi R. C. Zhu J. T. Leung K. W. Chu G. K. Xie H. Q. Chen V. P.. (2010). A flavonol glycoside, isolated from roots of Panax notoginseng, reduces amyloid-beta-induced neurotoxicity in cultured neurons: signaling transduction and drug development for Alzheimer’s disease. J. Alzheimers Dis. 19, 795–811. 10.3233/JAD-2010-1293 20157237

[B15] Choo M. K. Park E. K. Han M. J. Kim D. H. (2003). Antiallergic activity of ginseng and its ginsenosides. Planta Med. 69, 518–522. 10.1055/s-2003-40653 12865969

[B16] Chu S. Gu J. Feng L. Liu J. Zhang M. Jia X.. (2014). Ginsenoside Rg5 improves cognitive dysfunction and beta-amyloid deposition in STZ-induced memory impaired rats via attenuating neuroinflammatory responses. Int. Immunopharmacol. 19, 317–326. 10.1016/j.intimp.2014.01.018 24503167

[B17] Cui J. Jiang L. Xiang H. (2012). Ginsenoside Rb3 exerts antidepressant-like effects in several animal models. J. Psychopharmacol. 26, 697–713. 10.1177/0269881111415735 21948936

[B18] Dancey J. E. (2004). Molecular targeting: PI3 kinase pathway. Ann. Oncol. 15, iv233–iv239. 10.1093/annonc/mdh932 15477314

[B19] Dang H. Chen Y. Liu X. Wang Q. Wang L. Jia W.. (2009). Antidepressant effects of ginseng total saponins in the forced swimming test and chronic mild stress models of depression. Prog. Neuropsychopharmacol. Biol. Psychiatry 33, 1417–1424. 10.1016/j.pnpbp.2009.07.020 19632285

[B20] D’Angelo L. Grimaldi R. Caravaggi M. Marcoli M. Perucca E. Lecchini S.. (1986). A double-blind, placebo-controlled clinical study on the effect of a standardized ginseng extract on psychomotor performance in healthy volunteers. J. Ethnopharmacol. 16, 15–22. 10.1016/0378-8741(86)90063-2 3528672

[B21] Du X. Xu H. Jiang H. Xie J. (2013). Akt/Nrf2 activated upregulation of heme oxygenase-1 involves in the role of Rg1 against ferrous iron-induced neurotoxicity in SK-N-SH cells. Neurotox. Res. 24, 71–79. 10.1007/s12640-012-9362-3 23184650

[B22] Fang F. Chen X. Huang T. Lue L. F. Luddy J. S. Yan S. S. (2012). Multi-faced neuroprotective effects of Ginsenoside Rg1 in an Alzheimer mouse model. Biochim. Biophys. Acta 1822, 286–292. 10.1016/j.bbadis.2011.10.004 22015470 PMC3304026

[B23] Farooqui A. A. (2010). Neurochemical Aspects of Neurotraumatic and Neurodegenerative Diseases. New York: Springer.

[B24] Fu Y. Ji L. L. (2003). Chronic ginseng consumption attenuates age-associated oxidative stress in rats. J. Nutr. 133, 3603–3609. 14608081 10.1093/jn/133.11.3603

[B25] Fuzzati N. (2004). Analysis methods of ginsenosides. J. Chromatogr. B Analyt. Technol. Biomed. Life Sci. 812, 119–133. 10.1016/j.jchromb.2004.07.039 15556492

[B26] Gallagher S. (2004). Neurocognitive models of schizophrenia: a neurophenomenological critique. Psychopathology 37, 8–19. 10.1159/000077014 14988645

[B27] Gao Y. Chu S. F. Li J. P. Zhang Z. Yan J. Q. Wen Z. L.. (2015). Protopanaxtriol protects against 3-nitropropionic acid-induced oxidative stress in a rat model of Huntington’s disease. Acta Pharmacol. Sin. 36, 311–322. 10.1038/aps.2014.107 25640478 PMC4349920

[B28] González-Burgos E. Fernandez-Moriano C. Gómez-Serranillos M. P. (2014). Potential neuroprotective activity of Ginseng in Parkinson’s disease: a review. J. Neuroimmune. Pharmacol. 10, 14–29. 10.1007/s11481-014-9569-6 25349145

[B29] Hao K. Gong P. Sun S. Q. Hao H. P. Wang G. J. Dai Y.. (2011). Beneficial estrogen-like effects of ginsenoside Rb1, an active component of Panax ginseng, on neural 5-HT disposition and behavioral tasks in ovariectomized mice. Eur. J. Pharmacol. 659, 15–25. 10.1016/j.ejphar.2011.03.005 21414307

[B30] Hasegawa H. (2004). Proof of the mysterious efficacy of ginseng: basic and clinical trials: metabolic activation of ginsenoside: deglycosylation by intestinal bacteria and esterification with fatty acid. J. Pharmacol. Sci. 95, 153–157. 10.1254/jphs.fmj04001x4 15215638

[B32] Hasegawa H. Sung J. H. Benno Y. (1997). Role of human intestinal Prevotella oris in hydrolyzing ginseng saponins. Planta Med. 63, 436–440. 10.1055/s-2006-957729 9342949

[B31] Hasegawa H. Uchiyama M. (1998). Antimetastatic efficacy of orally administered ginsenoside Rb1 in dependence on intestinal bacterial hydrolyzing potential and significance of treatment with an active bacterial metabolite. Planta Med. 64, 696–700. 10.1055/s-2006-957560 9933987

[B33] He B. Chen P. Yang J. Yun Y. Zhang X. Yang R.. (2012). Neuroprotective effect of 20 (R)-ginsenoside Rg (3) against transient focal cerebral ischemia in rats. Neurosci. Lett. 526, 106–111. 10.1016/j.neulet.2012.08.022 22925661

[B34] He Q. Sun J. Wang Q. Wang W. He B. (2014). Neuroprotective effects of ginsenoside Rg1 against oxygen-glucose deprivation in cultured hippocampal neurons. J. Chin. Med. Assoc. 77, 142–149. 10.1016/j.jcma.2014.01.001 24548377

[B35] Heo J. H. Lee S. T. Chu K. Oh M. J. Park H. J. Shim J. Y.. (2008). An open-label trial of Korean red ginseng as an adjuvant treatment for cognitive impairment in patients with Alzheimer’s disease. Eur. J. Neurol. 15, 865–868. 10.1111/j.1468-1331.2008.02157.x 18684311

[B36] Heo J. H. Lee S. T. Oh M. J. Park H. J. Shim J. Y. Chu K.. (2011). Improvement of cognitive deficit in Alzheimer’s disease patients by long term treatment with korean red ginseng. J. Ginseng Res. 35, 457–461. 10.5142/jgr.2011.35.4.457 23717092 PMC3659550

[B37] Hou J. G. Xue J. J. Sun M. Q. Wang C. Y. Liu L. Zhang D. L.. (2012). Highly selective microbial transformation of major ginsenoside Rb1 to gypenoside LXXV by Esteya vermicola CNU120806. J. Appl. Microbiol. 113, 807–814. 10.1111/j.1365-2672.2012.05400.x 22805203

[B38] Hsieh W. T. Chiang B. H. (2014). A well-refined in vitro model derived from human embryonic stem cell for screening phytochemicals with midbrain dopaminergic differentiation-boosting potential for improving Parkinson’s disease. J. Agric. Food Chem. 62, 6326–6336. 10.1021/jf501640a 24933592

[B39] Huang J. Wu D. Wang J. Li F. Lu L. Gao Y.. (2014). Effects of Panax notoginseng saponin on alpha, beta and gamma secretase involved in Abeta deposition in SAMP8 mice. Neuroreport 25, 89–93. 10.1097/wnr.0000000000000048 24165110

[B40] Hwang S. H. Lee B. H. Choi S. H. Kim H. J. Jung S. W. Kim H. S.. (2015). Gintonin, a novel ginseng-derived lysophosphatidic acid receptor ligand, stimulates neurotransmitter release. Neurosci. Lett. 584, 356–361. 10.1016/j.neulet.2014.11.007 25445364

[B42] Hwang S. H. Shin T. J. Choi S. H. Cho H. J. Lee B. H. Pyo M. K.. (2012a). Gintonin, newly identified compounds from ginseng, is novel lysophosphatidic acids-protein complexes and activates G protein-coupled lysophosphatidic acid receptors with high affinity. Mol. Cells 33, 151–162. 10.1007/s10059-012-2216-z 22286231 PMC3887723

[B41] Hwang S. H. Shin E. J. Shin T. J. Lee B. H. Choi S. H. Kang J.. (2012b). Gintonin, a ginseng-derived lysophosphatidic acid receptor ligand, attenuates Alzheimer’s disease-related neuropathies: involvement of non-amyloidogenic processing. J. Alzheimers Dis. 31, 207–223. 10.3233/JAD-2012-120439 22543851

[B43] Itoh T. Zang Y. F. Murai S. Saito H. (1989). Effects of Panax ginseng root on the vertical and horizontal motor activities and on brain monoamine-related substances in mice. Planta Med. 55, 429–433. 10.1055/s-2006-962058 2813579

[B44] Jang S. Ryu J. H. Kim D. H. Oh S. (2004). Changes of [3H]MK-801, [3H]muscimol and [3H]flunitrazepam binding in rat brain by the prolonged ventricular infusion of transformed ginsenosides. Neurochem. Res. 29, 2257–2266. 10.1007/s11064-004-7034-2 15672548

[B45] Jellinger K. A. (2009). Recent advances in our understanding of neurodegeneration. J. Neural Transm. 116, 1111–1162. 10.1007/s00702-009-0240-y 19707851

[B46] Jeong H. G. Ko Y. H. Oh S. Y. Han C. Kim T. Joe S. H. (2014). Effect of Korean Red Ginseng as an adjuvant treatment for women with residual symptoms of major depression. Asia Pac. Psychiatry [Epub ahead of print]. 10.1111/appy.12169 25504813

[B47] Jia D. Deng Y. Gao J. Liu X. Chu J. Shu Y. (2014). Neuroprotective effect of Panax notoginseng plysaccharides against focal cerebral ischemia reperfusion injury in rats. Int. J. Biol. Macromol. 63, 177–180. 10.1016/j.ijbiomac.2013.10.034 24189392

[B49] Jiang Y. H. Li Y. B. Zhao X. Q. Chen D. Jiang R. Wang S. L. (2012a). [Effect of ginsenoside Rg1 on functional expression of human neural stem cells: a patch clamp study]. Zhongguo Zhong Yao Za Zhi 37, 3477–3480. 23373225

[B48] Jiang B. Xiong Z. Yang J. Wang W. Wang Y. Hu Z. L.. (2012b). Antidepressant-like effects of ginsenoside Rg1 are due to activation of the BDNF signalling pathway and neurogenesis in the hippocampus. Br. J. Pharmacol. 166, 1872–1887. 10.1111/j.1476-5381.2012.01902.x 22335772 PMC3402811

[B50] Jun Y. L. Bae C. H. Kim D. Koo S. Kim S. (2015). Korean Red Ginseng protects dopaminergic neurons by suppressing the cleavage of p35 to p25 in a Parkinson’s disease mouse model. J. Ginseng Res. 39, 148–154. 10.1016/j.jgr.2014.10.003 26045688 PMC4452523

[B51] Jung I. H. Lee J. H. Hyun Y. J. Kim D. H. (2012). Metabolism of ginsenoside Rb1 by human intestinal microflora and cloning of its metabolizing beta-D-glucosidase from Bifidobacterium longum H-1. Biol. Pharm. Bull. 35, 573–581. 10.1248/bpb.35.573 22466563

[B52] Jung J. S. Shin J. A. Park E. M. Lee J. E. Kang Y. S. Min S. W.. (2010). Anti-inflammatory mechanism of ginsenoside Rh1 in lipopolysaccharide-stimulated microglia: critical role of the protein kinase A pathway and hemeoxygenase-1 expression. J. Neurochem. 115, 1668–1680. 10.1111/j.1471-4159.2010.07075.x 20969575

[B53] Karpagam V. Sathishkumar N. Sathiyamoorthy S. Rasappan P. Shila S. Kim Y. J.. (2013). Identification of BACE1 inhibitors from Panax ginseng saponins-An Insilco approach. Comput. Biol. Med. 43, 1037–1044. 10.1016/j.compbiomed.2013.05.009 23816176

[B54] Keum Y. S. Han S. S. Chun K. S. Park K. K. Park J. H. Lee S. K.. (2003). Inhibitory effects of the ginsenoside Rg3 on phorbol ester-induced cyclooxygenase-2 expression, NF-kappaB activation and tumor promotion. Mutat. Res. 523–524, 75–85. 10.1016/s0027-5107(02)00323-8 12628505

[B55] Kiefer D. Pantuso T. (2003). Panax ginseng. Am. Fam. Physician 68, 1539–1542. 14596440

[B59] Kim S. Ahn K. Oh T. H. Nah S. Y. Rhim H. (2002). Inhibitory effect of ginsenosides on NMDA receptor-mediated signals in rat hippocampal neurons. Biochem. Biophys. Res. Commun. 296, 247–254. 10.1016/s0006-291x(02)00870-7 12163009

[B57] Kim J. Kim S. H. Lee D. S. Lee D. J. Kim S. H. Chung S.. (2013a). Effects of fermented ginseng on memory impairment and beta-amyloid reduction in Alzheimer’s disease experimental models. J. Ginseng Res. 37, 100–107. 10.5142/jgr.2013.37.100 23717163 PMC3659620

[B56] Kim E. H. Kim I. H. Lee M. J. Thach Nguyen C. Ha J. A. Lee S. C.. (2013b). Anti-oxidative stress effect of red ginseng in the brain is mediated by peptidyl arginine deiminase type IV (PADI4) repression via estrogen receptor (ER) beta up-regulation. J. Ethnopharmacol. 148, 474–485. 10.1016/j.jep.2013.04.041 23665163

[B60] Kim Y. M. Namkoong S. Yun Y. G. Hong H. D. Lee Y. C. Ha K. S.. (2007). Water extract of Korean red ginseng stimulates angiogenesis by activating the PI3K/Akt-dependent ERK1/2 and eNOS pathways in human umbilical vein endothelial cells. Biol. Pharm. Bull. 30, 1674–1679. 10.1248/bpb.30.1674 17827719

[B58] Kim M. S. Yu J. M. Kim H. J. Kim H. B. Kim S. T. Jang S. K.. (2014). Ginsenoside Re and Rd enhance the expression of cholinergic markers and neuronal differentiation in Neuro-2a cells. Biol. Pharm. Bull. 37, 826–833. 10.1248/bpb.b14-00011 24599032

[B65] Lee Y. J. Chung E. Lee K. Y. Lee Y. H. Huh B. Lee S. K. (1997). Ginsenoside-Rg1, one of the major active molecules from Panax ginseng, is a functional ligand of glucocorticoid receptor. Mol. Cell. Endocrinol. 133, 135–140. 10.1016/s0303-7207(97)00160-3 9406859

[B62] Lee K. J. Ji G. E. (2014). The effect of fermented red ginseng on depression is mediated by lipids. Nutr. Neurosci. 17, 7–15. 10.1179/1476830513y.0000000059 24088416

[B61] Lee J. Jo D. G. Park D. Chung H. Y. Mattson M. P. (2014). Adaptive cellular stress pathways as therapeutic targets of dietary phytochemicals: focus on the nervous system. Pharmacol. Rev. 66, 815–868. 10.1124/pr.113.007757 24958636 PMC4081729

[B63] Lee K. W. Jung S. Y. Choi S. M. Yang E. J. (2012). Effects of ginsenoside Re on LPS-induced inflammatory mediators in BV2 microglial cells. BMC Complement. Altern. Med. 12:196. 10.1186/1472-6882-12-196 23102375 PMC3517379

[B64] Lee S. Y. Kim G. T. Roh S. H. Song J. S. Kim H. J. Hong S. S.. (2009). Proteomic analysis of the anti-cancer effect of 20S-ginsenoside Rg3 in human colon cancer cell lines. Biosci. Biotechnol. Biochem. 73, 811–816. 10.1271/bbb.80637 19352032

[B66] Lee Y. Y. Park J. S. Lee E. J. Lee S. Y. Kim D. H. Kang J. L.. (2015). Anti-inflammatory mechanism of ginseng saponin metabolite Rh3 in lipopolysaccharide-stimulated microglia: critical role of 5’-adenosine monophosphate-activated protein kinase signaling pathway. J. Agric. Food Chem. 63, 3472–3480. 10.1021/jf506110y 25798758

[B67] Leung K. W. Cheng Y. K. Mak N. K. Chan K. K. Fan T. P. Wong R. N. (2006). Signaling pathway of ginsenoside-Rg1 leading to nitric oxide production in endothelial cells. FEBS Lett. 580, 3211–3216. 10.1016/j.febslet.2006.04.080 16696977

[B69] Li M. Ling C. Q. Huang X. Q. Shen Z. L. (2006). [Effects of ginsenosides extracted from ginseng stem and leaves on glucocorticoid receptor in different viscera in heat-damaged rats]. Zhong Xi Yi Jie He Xue Bao 4, 156–159. 10.3736/jcim20060210 16529692

[B68] Li L. Liu J. Yan X. Qin K. Shi M. Lin T.. (2011a). Protective effects of ginsenoside Rd against okadaic acid-induced neurotoxicity in vivo and in vitro. J. Ethnopharmacol. 138, 135–141. 10.1016/j.jep.2011.08.068 21945003

[B70] Li Y. Tang J. Khatibi N. H. Zhu M. Chen D. Tu L.. (2011b). Treatment with ginsenoside rb1, a component of panax ginseng, provides neuroprotection in rats subjected to subarachnoid hemorrhage-induced brain injury. Acta Neurochir. Suppl. 110, 75–79. 10.1007/978-3-7091-0356-2_14 21125449

[B71] Li Y. Tang J. Khatibi N. H. Zhu M. Chen D. Zheng W.. (2010). Ginsenoside Rbeta1 reduces neurologic damage, is anti-apoptotic and down-regulates p53 and BAX in subarachnoid hemorrhage. Curr. Neurovasc. Res. 7, 85–94. 10.2174/156720210791184952 20353383

[B72] Li Y. B. Zhao X. Q. Jiang Y. H. Chen D. Wang S. L. (2013). [Study on molecular target promoting human neural stem cells of ginsenoside Rg1 by gene chip]. Zhongguo Zhong Yao Za Zhi 38, 2701–2705. 10.4268/cjcmm20131631 24228590

[B74] Lin Z. Y. Chen L. M. Zhang J. Pan X. D. Zhu Y. G. Ye Q. Y.. (2012a). Ginsenoside Rb1 selectively inhibits the activity of L-type voltage-gated calcium channels in cultured rat hippocampal neurons. Acta Pharmacol. Sin. 33, 438–444. 10.1038/aps.2011.181 22407229 PMC4003359

[B73] Lin T. Liu Y. Shi M. Liu X. Li L. Liu Y.. (2012b). Promotive effect of ginsenoside Rd on proliferation of neural stem cells in vivo and in vitro. J. Ethnopharmacol. 142, 754–761. 10.1016/j.jep.2012.05.057 22683911

[B77] Liu L. Hoang-Gia T. Wu H. Lee M. R. Gu L. Wang C.. (2011). Ginsenoside Rb1 improves spatial learning and memory by regulation of cell genesis in the hippocampal subregions of rats. Brain Res. 1382, 147–154. 10.1016/j.brainres.2011.01.051 21276426

[B76] Liu H. Yang J. Du F. Gao X. Ma X. Huang Y.. (2009). Absorption and disposition of ginsenosides after oral administration of Panax notoginseng extract to rats. Drug Metab. Dispos. 37, 2290–2298. 10.1124/dmd.109.029819 19786509

[B75] Liu D. Zhang H. Gu W. Liu Y. Zhang M. (2013). Neuroprotective effects of ginsenoside Rb1 on high glucose-induced neurotoxicity in primary cultured rat hippocampal neurons. PLoS One 8:e79399. 10.1371/journal.pone.0079399 24223941 PMC3815219

[B79] Liu Z. J. Zhao M. Zhang Y. Xue J. F. Chen N. H. (2010). Ginsenoside Rg1 promotes glutamate release via a calcium/calmodulin-dependent protein kinase II-dependent signaling pathway. Brain Res. 1333, 1–8. 10.1016/j.brainres.2010.03.096 20381470

[B78] Liu L. Zhu L. Zou Y. Liu W. Zhang X. Wei X.. (2014). Panax notoginseng saponins promotes stroke recovery by influencing expression of Nogo-A, NgR and p75NGF, in vitro and in vivo. Biol. Pharm. Bull. 37, 560–568. 10.1248/bpb.b13-00770 24818251

[B80] Luo F. C. Wang S. D. Qi L. Song J. Y. Lv T. Bai J. (2011). Protective effect of panaxatriol saponins extracted from Panax notoginseng against MPTP-induced neurotoxicity in vivo. J. Ethnopharmacol. 133, 448–453. 10.1016/j.jep.2010.10.017 20951784

[B81] Meng X. Sun G. Ye J. Xu H. Wang H. Sun X. (2014). Notoginsenoside R1-mediated neuroprotection involves estrogen receptor-dependent crosstalk between Akt and ERK1/2 pathways: a novel mechanism of Nrf2/ARE signaling activation. Free Radic. Res. 48, 445–460. 10.3109/10715762.2014.885117 24437944

[B82] Nah S. Y. (2012). Gintonin: a novel ginseng-derived ligand that targets G protein- coupled lysophosphatidic acid receptors. Curr. Drug Targets 13, 1659–1664. 10.2174/138945012803529947 23017203

[B83] Nah S. Y. Kim D. H. Rhim H. (2007). Ginsenosides: are any of them candidates for drugs acting on the central nervous system? CNS Drug Rev. 13, 381–404. 18078425 10.1111/j.1527-3458.2007.00023.x PMC6494168

[B84] Park H. Kim S. Rhee J. Kim H. J. Han J. S. Nah S. Y.. (2015). Synaptic enhancement induced by gintonin via lysophosphatidic acid receptor activation in central synapses. J. Neurophysiol. 113, 1493–1500. 10.1152/jn.00667.2014 25505112

[B85] Park J. S. Shin J. A. Jung J. S. Hyun J. W. Van Le T. K. Kim D. H.. (2012a). Anti-inflammatory mechanism of compound K in activated microglia and its neuroprotective effect on experimental stroke in mice. J. Pharmacol. Exp. Ther. 341, 59–67. 10.1124/jpet.111.189035 22207656

[B86] Park S. M. Choi M. S. Sohn N. W. Shin J. W. (2012b). Ginsenoside Rg3 attenuates microglia activation following systemic lipopolysaccharide treatment in mice. Biol. Pharm. Bull. 35, 1546–1552. 10.1248/bpb.b12-00393 22975507

[B87] Pilpel Y. Segal M. (2006). The role of LPA1 in formation of synapses among cultured hippocampal neurons. J. Neurochem. 97, 1379–1392. 10.1111/j.1471-4159.2006.03825.x 16638019

[B88] Qi X. Ignatova S. Luo G. Liang Q. Jun F. W. Wang Y.. (2010). Preparative isolation and purification of ginsenosides Rf, Re, Rd and Rb1 from the roots of Panax ginseng with a salt/containing solvent system and flow step-gradient by high performance counter-current chromatography coupled with an evaporative light scattering detector. J. Chromatogr. A 1217, 1995–2001. 10.1016/j.chroma.2010.01.057 20171644

[B89] Qian T. Jiang Z. H. Cai Z. (2006). High-performance liquid chromatography coupled with tandem mass spectrometry applied for metabolic study of ginsenoside Rb1 on rat. Anal. Biochem. 352, 87–96. 10.1016/j.ab.2006.02.025 16564485

[B90] Qiu J. Li W. Feng S. H. Wang M. He Z. Y. (2014). Ginsenoside Rh2 promotes nonamyloidgenic cleavage of amyloid precursor protein via a cholesterol-dependent pathway. Genet. Mol. Res. 13, 3586–3598. 10.4238/2014.may.9.2 24854439

[B91] Radad K. Moldzio R. Rausch W. D. (2011). Ginsenosides and their CNS targets. CNS Neurosci. Ther. 17, 761–768. 10.1111/j.1755-5949.2010.00208.x 21143430 PMC6493809

[B92] Rudakewich M. Ba F. Benishin C. G. (2001). Neurotrophic and neuroprotective actions of ginsenosides Rb (1) and Rg (1). Planta Med. 67, 533–537. 10.1055/s-2001-16488 11509974

[B93] Seidl S. E. Santiago J. A. Bilyk H. Potashkin J. A. (2014). The emerging role of nutrition in Parkinson’s disease. Front. Aging Neurosci. 6:36. 10.3389/fnagi.2014.00036 24639650 PMC3945400

[B94] Shi S. Shi R. Hashizume K. (2012). American ginseng improves neurocognitive function in senescence-accelerated mice: possible role of the upregulated insulin and choline acetyltransferase gene expression. Geriatr. Gerontol. Int. 12, 123–130. 10.1111/j.1447-0594.2011.00719.x 21702872

[B95] Shin H. R. Kim J. Y. Yun T. K. Morgan G. Vainio H. (2000). The cancer-preventive potential of Panax ginseng: a review of human and experimental evidence. Cancer Causes Control 11, 565–576. 10.1023/A:1008980200583 10880039

[B96] Sohn S. H. Kim S. K. Kim Y. O. Kim H. D. Shin Y. S. Yang S. O.. (2013). A comparison of antioxidant activity of Korean White and Red Ginsengs on H2O2-induced oxidative stress in HepG2 hepatoma cells. J. Ginseng Res. 37, 442–450. 10.5142/jgr.2013.37.442 24233437 PMC3825859

[B98] Song X. Y. Hu J. F. Chu S. F. Zhang Z. Xu S. Yuan Y. H.. (2013). Ginsenoside Rg1 attenuates okadaic acid induced spatial memory impairment by the GSK3βtau signaling pathway and the Abeta formation prevention in rats. Eur. J. Pharmacol. 710, 29–38. 10.1016/j.ejphar.2013.03.051 23588117

[B97] Song C. S. Shi Y. Song J. Tian J. J. Guo J. Z. Dai J. X. (2006). Effect of qutan huoluo jiannao preparation in improving memory impairment of rats with cerebral ischemia. Chin. J. Clin. Rehabil. 10, 32–35.

[B99] Tang B. Qu Y. Wang D. Mu D. (2011). Targeting hypoxia inducible factor-1alpha: a novel mechanism of ginsenoside Rg1 for brain repair after hypoxia/ischemia brain damage. CNS Neurol. Disord. Drug Targets 10, 235–238. 10.2174/187152711794480456 20874696

[B100] Tawab M. A. Bahr U. Karas M. Wurglics M. Schubert-Zsilavecz M. (2003). Degradation of ginsenosides in humans after oral administration. Drug Metab. Dispos. 31, 1065–1071. 10.1124/dmd.31.8.1065 12867496

[B101] Tu L. H. Ma J. Liu H. P. Wang R. R. Luo J. (2009). The neuroprotective effects of ginsenosides on calcineurin activity and tau phosphorylation in SY5Y cells. Cel. Mol. Neurobiol. 29, 1257–1264. 10.1007/s10571-009-9421-3 19517226 PMC11505759

[B103] Van Kampen J. M. Baranowski D. B. Shaw C. A. Kay D. G. (2014). Panax ginseng is neuroprotective in a novel progressive model of Parkinson’s disease. Exp. Gerontol. 50, 95–105. 10.1016/j.exger.2013.11.012 24316034

[B102] Van Kampen J. Robertson H. Hagg T. Drobitch R. (2003). Neuroprotective actions of the ginseng extract G115 in two rodent models of Parkinson’s disease. Exp. Neurol. 184, 521–529. 10.1016/j.expneurol.2003.08.002 14637121

[B108] Wang Y. Feng Y. Fu Q. Li L. (2013a). Panax notoginsenoside Rb1 ameliorates Alzheimer’s disease by upregulating brain-derived neurotrophic factor and downregulating Tau protein expression. Exp. Ther. Med. 6,826–830. 24137274 10.3892/etm.2013.1215 PMC3786787

[B104] Wang B. Feng G. Tang C. Wang L. Cheng H. Zhang Y.. (2013b). Ginsenoside Rd maintains adult neural stem cell proliferation during lead-impaired neurogenesis. Neurol. Sci. 34, 1181–1188. 10.1007/s10072-012-1215-6 23073826

[B105] Wang C. M. Liu M. Y. Wang F. Wei M. J. Wang S. Wu C. F.. (2013c). Anti-amnesic effect of pseudoginsenoside-F11 in two mouse models of Alzheimer’s disease. Pharmacol. Biochem. Behav. 106, 57–67. 10.1016/j.pbb.2013.03.010 23541491

[B109] Wang Y. Liu J. Zhang Z. Bi P. Qi Z. Zhang C. (2011a). Anti-neuroinflammation effect of ginsenoside Rbl in a rat model of Alzheimer disease. Neurosci. Lett. 487, 70–72. 10.1016/j.neulet.2010.09.076 20933058

[B110] Wang Z. J. Sun L. Peng W. Ma S. Zhu C. Fu F.. (2011b). Ginseng derivative ocotillol enhances neuronal activity through increased glutamate release: a possible mechanism underlying increased spontaneous locomotor activity of mice. Neuroscience 195, 1–8. 10.1016/j.neuroscience.2011.08.002 21864652 PMC3193848

[B106] Wang H. Peng D. Xie J. (2009). Ginseng leaf-stem: bioactive constituents and pharmacological functions. Chin. Med. 4:20. 10.1186/1749-8546-4-20 19849852 PMC2770043

[B107] Wang W. Zhao Y. Rayburn E. R. Hill D. L. Wang H. Zhang R. (2007). In vitro anti-cancer activity and structure-activity relationships of natural products isolated from fruits of Panax ginseng. Cancer Chemother. Pharmacol. 59, 589–601. 10.1007/s00280-006-0300-z 16924497

[B111] Wiklund I. K. Mattsson L. A. Lindgren R. Limoni C. (1999). Effects of a standardized ginseng extract on quality of life and physiological parameters in symptomatic postmenopausal women: a double-blind, placebo-controlled trial. Swedish alternative medicine group. Int. J. Clin. Pharmacol. Res. 19, 89–99. 10761538

[B112] Xiang H. Liu Y. Zhang B. Huang J. Li Y. Yang B.. (2011). The antidepressant effects and mechanism of action of total saponins from the caudexes and leaves of Panax notoginseng in animal models of depression. Phytomedicine 18, 731–738. 10.1016/j.phymed.2010.11.014 21273053

[B113] Xu C. Teng J. Chen W. Ge Q. Yang Z. Yu C.. (2010). 20 (S)-protopanaxadiol, an active ginseng metabolite, exhibits strong antidepressant-like effects in animal tests. Prog. Neuropsychopharmacol. Biol. Psychiatry 34, 1402–1411. 10.1016/j.pnpbp.2010.07.010 20647027

[B114] Xu T. M. Xin Y. Cui M. H. Jiang X. Gu L. P. (2007). Inhibitory effect of ginsenoside Rg3 combined with cyclophosphamide on growth and angiogenesis of ovarian cancer. Chin. Med. J. (Engl) 120, 584–588. 17442207

[B115] Xue J. F. Liu Z. J. Hu J. F. Chen H. Zhang J. T. Chen N. H. (2006). Ginsenoside Rb1 promotes neurotransmitter release by modulating phosphorylation of synapsins through a cAMP-dependent protein kinase pathway. Brain Res. 1106, 91–98. 10.1016/j.brainres.2006.05.106 16836988

[B117] Yan S. Li Z. Li H. Arancio O. Zhang W. (2014). Notoginsenoside R1 increases neuronal excitability and ameliorates synaptic and memory dysfunction following amyloid elevation. Sci. Rep. 4:6352. 10.1038/srep06352 25213453 PMC4161968

[B116] Yan J. Liu Q. Dou Y. Hsieh Y. Liu Y. Tao R.. (2013). Activating glucocorticoid receptor-ERK signaling pathway contributes to ginsenoside Rg1 protection against beta-amyloid peptide-induced human endothelial cells apoptosis. J. Ethnopharmacol. 147, 456–466. 10.1016/j.jep.2013.03.039 23538162

[B118] Yang L. Zhang J. Zheng K. Shen H. Chen X. (2014). Long-term ginsenoside Rg1 supplementation improves age-related cognitive decline by promoting synaptic plasticity associated protein expression in C57BL/6J mice. J. Gerontol. A Biol. Sci. Med. Sci. 69, 282–294. 10.1093/gerona/glt091 23833204

[B119] Ye R. Kong X. Yang Q. Zhang Y. Han J. Li P.. (2011a). Ginsenoside rd in experimental stroke: superior neuroprotective efficacy with a wide therapeutic window. Neurotherapeutics 8, 515–525. 10.1007/s13311-011-0051-3 21647765 PMC3250281

[B120] Ye R. Yang Q. Kong X. Han J. Zhang X. Zhang Y.. (2011b). Ginsenoside Rd attenuates early oxidative damage and sequential inflammatory response after transient focal ischemia in rats. Neurochem. Int. 58, 391–398. 10.1016/j.neuint.2010.12.015 21185898

[B121] Yoon M. Lee H. Jeong S. Kim J. J. Nicol C. J. Nam K. W.. (2003). Peroxisome proliferator-activated receptor alpha is involved in the regulation of lipid metabolism by ginseng. Br. J. Pharmacol. 138, 1295–1302. 10.1038/sj.bjp.0705169 12711630 PMC1573779

[B122] Yue P. Y. Wong D. Y. Wu P. K. Leung P. Y. Mak N. K. Yeung H. W.. (2006). The angiosuppressive effects of 20 (R)- ginsenoside Rg3. Biochem. Pharmacol. 72, 437–445. 10.1016/j.bcp.2006.04.034 16793023

[B124] Zhang Y. Lin L. Liu G. Y. Liu J. X. Li T. (2014). [Pharmacokinetics and brain distribution of ginsenosides after administration of sailuotong]. Zhongguo Zhong Yao Za Zhi 39, 316–321. 10.4268/cjcmm20140230 24761653

[B123] Zhang X. Shi M. Bjørås M. Wang W. Zhang G. Han J.. (2013). Ginsenoside Rd promotes glutamate clearance by up-regulating glial glutamate transporter GLT-1 via PI3K/AKT and ERK1/2 pathways. Front. Pharmacol. 4:152. 10.3389/fphar.2013.00152 24376419 PMC3858668

[B125] Zhang Y. Zhou L. Zhang X. Bai J. Shi M. Zhao G. (2012). Ginsenoside-Rd attenuates TRPM7 and ASIC1a but promotes ASIC2a expression in rats after focal cerebral ischemia. Neurol. Sci. 33, 1125–1131. 10.1007/s10072-011-0916-6 22231470

[B127] Zhao H. H. Di J. Liu W. S. Liu H. L. Lai H. üu Y. L. (2013). Involvement of GSK3 and PP2A in ginsenoside Rb1’s attenuation of aluminum-induced tau hyperphosphorylation. Behav. Brain Res. 241, 228–234. 10.1016/j.bbr.2012.11.037 23219964

[B128] Zhao X. Gao J. Song C. Fang Q. Wang N. Zhao T.. (2012). Fungal sensitivity to and enzymatic deglycosylation of ginsenosides. Phytochemistry 78, 65–71. 10.1016/j.phytochem.2012.02.027 22449289

[B126] Zhao H. F. Li Q. Li Y. (2011). Long-term ginsenoside administration prevents memory loss in aged female C57BL/6J mice by modulating the redox status and up-regulating the plasticity-related proteins in hippocampus. Neuroscience 183, 189–202. 10.1016/j.neuroscience.2011.03.048 21463662

[B129] Zheng Y. Q. Liu J. X. Li X. Z. Xu L. (2010). Effects and mechanism of Weinaokang on reperfusion-induced vascular injury to cerebral microvessels after global cerebral ischemia. Chin. J. Integr. Med. 16, 145–150. 10.1007/s11655-010-0145-5 20473740

[B130] Zhong Z. G. Lv L. Chai L. M. Wu D. P. Zhang W. Y. Huang J. L.. (2011). [Effect of Panax notoginseng saponins on APP gene transcription in the brain tissue of SAMP8]. Zhong Yao Cai 34, 77–80. 21818973

[B132] Zhou Y. Li H. Q. Lu L. Fu D. L. Liu A. J. Li J. H.. (2014a). Ginsenoside Rg1 provides neuroprotection against blood brain barrier disruption and neurological injury in a rat model of cerebral ischemia/reperfusion through downregulation of aquaporin 4 expression. Phytomedicine 21, 998–1003. 10.1016/j.phymed.2013.12.005 24462216

[B131] Zhou N. Tang Y. Keep R. F. Ma X. Xiang J. (2014b). Antioxidative effects of Panax notoginseng saponins in brain cells. Phytomedicine 21, 1189–1195. 10.1016/j.phymed.2014.05.004 24916704 PMC4111966

[B134] Zhu J. Jiang Y. Wu L. Lu T. Xu G. Liu X. (2012). Suppression of local inflammation contributes to the neuroprotective effect of ginsenoside Rb1 in rats with cerebral ischemia. Neuroscience 202, 342–351. 10.1016/j.neuroscience.2011.11.070 22173011

[B133] Zhu D. Liu M. Yang Y. Ma L. Jiang Y. Zhou L.. (2014). Ginsenoside Rd ameliorates experimental autoimmune encephalomyelitis in C57BL/6 mice. J. Neurosci. Res. 92, 1217–1226. 10.1002/jnr.23397 24798871

[B135] Zhu S. Zou K. Cai S. Meselhy M. R. Komatsu K. (2004). Simultaneous determination of triterpene saponins in ginseng drugs by high-performance liquid chromatography. Chem. Pharm. Bull (Tokyo) 52, 995–998. 10.1248/cpb.52.995 15305000

[B136] Zong Y. Ai Q. L. Zhong L. M. Dai J. N. Yang P. He Y.. (2012). Ginsenoside Rg1 attenuates lipopolysaccharide-induced inflammatory responses via the phospholipase C-gamma1 signaling pathway in murine BV-2 microglial cells. Curr. Med. Chem. 19, 770–779. 10.2174/092986712798992066 22214447

